# Diphtheria : Some Points in the Pathology and Treatment

**Published:** 1894-01-27

**Authors:** Arnold W. W. Lea

**Affiliations:** late Senior Resident Medical Officer to the Childrens' Hospital, Pendlebury


### DIPHTHERIA: SOME POINTS IN THE PATHOLOGY AND TREATMENT.

By Arnold W. W. Lea, M.D., B.S. (Lond.), F.R.C.S. (Eng.), late Senior Resident Medical Officer to the Childrens' Hospital, Pendlebury.

The researches of the last few years have greatly increased our knowledge of the pathology of this affection, and have also given us very important aid, both in the diagnosis and treatment. Diphtheria has long been supposed to be due to some micro-organism, and several, supposed to be the cause of it, have been described.

The bacillus of Loeffler is now regarded on very good grounds as being the immediate cause of the disease. The bacillus enters usually by the mouth or nose, and is deposited in the throat or larynx; if it here finds a suitable nidus it grows, and as a result acute inflammation of the mucous membrane is set up, which causes a fibrinous exudation, the diphtheritic membrane. These bacilli are very tenacious of life; they thrive outside the body, and retain their vitality on clothes, articles of furniture, and walls of rooms for an indefinite time; they also develope readily in milk. If a portion of diphtheritic membrane be examined microscopically, it is seen to consist of fibrinous exudation with leucocytes and epithelial cells, and near the surface of the membrane typical bacilli will be found. It is very important to remember that the bacilli do not penetrate into living tissue, but remain limited to the superficial part of the membrane. How then are the general symptoms of diphtheria produced?

Dr. Sidney Martin has recently investigated this subject from a chemical as well as a bacteriological standpoint. He has examined the blood and the spleen of a number of diphtheria cases after death. In all these cases the presence of the Loeffler bacillus had been ascertained during life. From the blood he was able to extract a powerful poison, which inoculated into rabbits produced the toxic symptoms of diphtheria, especially the paralysis, but did not cause any formation of membrane. His conclusions are as follows: The bacilli produce during their growth some substance which acts on the fibrinous membrane, and causes the formation of a soluble albumose, together with a material of the nature of a ferment. These are absorbed into the general circulation, and cause the well-known symptoms of anæmia, albuminuria, cardiac weakness, and paralysis. It is, of course, extremely important that a case of diphtheria should be diagnosed as early as possible. From the examination of the throat it is frequently impossible to say definitely whether the case be true diphtheria or not, and in cases of laryngeal diphtheria, so frequent in children, no membrane can be seen, and none may be coughed up. In these cases the detection of the bacilli is the only certain test in the early stages when diagnosis is so important. Later on, of course, when the typical symptoms develope diagnosis is easy. The method of procedure is briefly as follows: If any membrane can be seen remove a small piece with sterilised forceps, and place it rapidly in a sterilised blood-serum cultivating test-tube; if no membrane can be obtained a little of the mucus from the throat may be taken and streaked in a test-tube with a platinum wire, previously sterilised by heat. The tube is put into an incubator, and kept at a temperature of 38 deg. C. If the bacilli are present, within twenty-four hours a typical growth will be obtained, and they may be recognised microscopically. In many cases the bacilli may be discovered in the mucus of the throat or in a piece of membrane without making a cultivation, though this method is not so reliable.

Numerous micrococci are found in the throat in diphtheria, in addition to the bacilli, and in the forms attended by great enlargement of lymphatic glands and suppuration the streptococcus pyogenes is always found abundantly, and is probably the cause of the severe inflammatory symptoms.

It is quite possible to have diphtheria without any membrane being recognised upon the throat or larynx during life or even after death. In one case quoted by Dr. Martin, a child died of croup 24 hours after tracheotomy had been performed. No membrane was seen during life, and after death no membrane was visible to the naked eye. A few small erosions were observed about the epiglottis and upper orifice of the larynx; microscopically a few shreds of membrane were seen on the erosions, and numerous bacilli were found. In this case Dr. Martin found a large amount of diphtheritic poison in the blood, so that the toxic effects do not seem to depend entirely on the amount of membranous exudation. The diagnosis of diphtheria from some other affections of the throat and larynx may be very difficult. In practice it may be quite impossible to differentiate between acute catarrhal laryngitis and diphtheritic laryngitis in children, at least in the early stages, and even after tracheotomy the diagnosis may remain obscure. Again in some cases of mild faucial diphtheria, when the exudation is limited to one small patch which soon disappears, the diagnosis may be uncertain, in the absence of the general symptoms of diphtheria.

It is in these cases that the examination for the bacilli proves extremely useful.

As regards diphtheria of the larynx and membranous croup, most authorities now agree that nearly all cases of membranous croup are true diphtheria. In some exceptional cases, however, false membrane forms upon the larynx, as a result of scalds of the throat, and also in the acute infective diseases, such as measles and scarlet fever.

*Treatment.*—It is evident from what we have seen that diphtheria is, in the first instance, a purely local disease, and due to the invasion of a mucous surface, most commonly on the throat or larynx, by the specific bacilli. The constitutional symptoms are due to the development of certain toxic substances which enter the general circulation. It is thus evident that we now have something definite to combat by our treatment.

Firstly, as regards preventive treatment. Diphtheria is contagious; the breath of a diphtheria patient is probably not infectious, but the danger lies in the secretion from the throat and in the particles of membrane, which may be conveyed in many ways to those in attendance on the patients, and also to utensils and articles of furniture, bedding, &c.

Now the bacilli, even if they effect a lodgment on a mucous surface, cannot develop unless they find a suitable nidus. If the throat and larynx are perfectly healthy, and the subject has good powers of resistance, it is probable that the bacilli are rapidly destroyed by the action of the leucocytes before they are able to proliferate.

Hence cases of diphtheria should be completely isolated. The sick room should be large and well ventilated. Those in attendance should exercise the greatest care in thoroughly disinfecting all articles used by the patient, and should also see that none of the secretion from the throat or nose is allowed to remain in the room. The discharge should be wiped away with soft linen or lint, which can be immediately burnt. Any membrane which is coughed up or removed should be burnt at once.

It is, perhaps, well for those in constant attendance on diphtheria cases to gargle the throat with a solution of perchloride of mercury (1-10,000) every three or four hours, as a preventive of infection. The best means of checking the spread of diphtheria are, however, abundance of fresh air, sunlight, and perfect sanitary conditions. It is a disease which continually occurs in insanitary and badly drained dwellings, though cases often arise in houses where these conditions do not exist.

In the treatment there are two main factors to be considered. In the first place, the bacilli which accumulate in the membrane must be destroyed, since they develope in their growth the toxic agents which so frequently cause a fatal termination. In the second place we must try to prevent the spread of the bacilli into surrounding healthy tissue. Caustic applications to the affected parts are very likely to do harm, for they frequently cause injury to the surrounding healthy mucous membrane, thus lowering its vitality, and so favouring the spread of mischief.

Loeffler has found experimentally that perchloride of mercury is most effectual in destroying the bacilli, and I have treated a considerable number of cases of pharyngeal diphtheria with this drug used locally.

The throat should first be cleansed by syringing with some antiseptic solution of a non-poisonous nature, such as tincture of iodine (1-40), Condy's fluid (1-40), or liquor sodæ chlorinatæ (1-20). If there is any discharge from the nose this should also be similarly syringed. After the throat is cleansed of all mucus, decomposing food, and any particles of loose membrane, an application should be made. For this, a solution of perchloride of mercury, in glycerine (1-500) is probably as effectual as any. This may be applied by a brush, or preferably by absorbent wool, firmly tied round a piece of stick, and then the wool can be burnt immediately after use. I have never seen any symptoms of mercurial poisoning from this treatment, and the results are fairly satisfactory, though it is impossible to speak very dogmatically from the experience of a limited number of cases. Very many other applications are used and recommended by different authorities, such as carbolic acid and glycerine, boroglyceride, salicylic acid and glycerine, and many others.

In the early stage, when only one or two patches are seen, the galvano-cautery has been used, the membrane is burnt off and is said not to form again. The injection of liquor sodæ chlorinatæ into the sub-membranous tissue, by means of a special syringe, has been strongly recommended by Siebert. I have no experience of these methods of treatment. It must be acknowledged that whatever method is used, in a certain number of cases of pharyngeal diphtheria the disease spreads to the nose or larynx, and these cases often prove fatal.

Whatever application is decided upon should be used thoroughly. The patient, if a child, should be firmly held by the nurse, the mouth must be opened, avoiding any force as far as possible, and the throat cleansed by thorough syringing. The application should then be made. The frequency with which this should be done depends on the severity of the disease; every three or four hours is usually often enough. If the patient is old enough the throat may be sprayed with any of the above solutions, or with a solution of perchloride of mercury, 1-10,000.

Any loose membrane should be removed by syringing or forceps, but it is not advisable to detach it forcibly, as fresh membrane readily re forms on the raw surface.

If the membrane extends to the larynx, as shown by difficult breathing, croupy cough, and signs of laryngeal obstruction, the case, however mild previously, at once becomes very serious.

If the obstruction to the entry of air is considerable, tracheotomy or intubation will almost certainly be required, and should be done early before signs of exhaustion set in. These operations always relieve the breathing for a time, and many cases recover completely; but if, as frequently occurs, the membrane spreads to the trachea and bronchi, the patients generally die.

It is impossible, however, in a short paper of this kind to enter into the details of the treatment of this class of case.

In the general treatment we have to combat the toxic effects of the poison in the blood. Unfortunately, no drug is known at present which has any specific effect. There is no evidence to show that the administration of antiseptics, such as carbolic acid or the sulpho-carbolates, has any direct effect on the toxæmia. Probably the tinct. ferri. perchlor. in five minim doses every four hours is the best medicine, as it tends to counteract the intense anæmia so often seen. If signs of cardiac failure manifest themselves digitalis should be given with ammonia and ether.

The feeding is very important. Nourishing liquid food must be given in abundance, and at frequent intervals, milk, strong beef-tea, or one of the beef extracts. Benger's peptonised beef jelly is often very valuable and readily digested. Alcohol should be given freely, probably brandy or port wine are the best forms to administer. There is often great difficulty in getting patients to swallow their food well on account of the pain and swelling of the throat. Food may then if necessary be administered by a tube through the mouth or nose, though this should be avoided, if possible, as the patient often struggles and exhausts himself, and also injury may be done to the inflamed parts.

Rectal feeding with peptonised beef tea and eggs, or nutrient suppositories is sometimes advantageous.

If the case does well, in so much that the membrane ceases to form, and clears up, our anxieties are by no means over. Applications to the throat should be continued for some time, as there is good reason to think that the bacilli may remain on the throat after the membrane has disappeared. The patient must be kept in bed, and any sudden exertion avoided, since the heart is always feeble, and there is great risk of syncope. In one case which I saw recently the child was doing well, and the throat had been clear of membrane for some days; the mother, however, administered an enema and allowed the child to sit up. As a result cardiac failure occurred, and when the child was seen a few minutes later no pulse could be felt and the heart sounds were almost inaudible; fortunately, under the influence of stimulants, the child rallied and recovered completely.

Many cases are followed by paralysis, which may come on any time within five or six weeks of the onset of the disease. This is usually slight and affects specially the soft palate, causing regurgitation of liquid through the nose on swallowing fluid. It may, however, become very extensive, reducing the patient to a condition of complete helplessness. Most cases recover completely, so long as the respiratory muscles are not paralysed. It is probably best treated by strychnine and iron, with massage and electricity. In bad cases strychnine should be injected hypodermically. The albuminuria of diphtheria, though very constant, does not often call for special treatment. If large in amount it renders the prognosis grave. In many cases little urine is passed during the later stages of the disease, but this is more often due to the low blood pressure and asthenic condition than to suppression of urine from inflammatory mischief. Uræmia is very uncommon. Bright's disease also rarely results after diphtheria.

